# Combination Technique of Radiofrequency Ablation with Sclerotherapy in Acquired Lymphangiectasis of the Vulva

**DOI:** 10.4103/0974-2077.53098

**Published:** 2009

**Authors:** Niti Khunger

**Affiliations:** *Department of Dermatology, VM Medical College and Safdarjang Hospital, New Delhi, India*

**Keywords:** Radiofrequency ablation, sclerotherapy, vulvar lymphangiectasis

## Abstract

Aquired lymphangiectasis of the vulva is an uncommon condition and only few cases have been reported following tuberculous lymphadenitis. A case is reported that was successfully treated with a unique combination therapy of radiofrequency ablation and sclerotherapy with polidocanol. There was no recurrence at two years of follow-up.

## INTRODUCTION

Lymphangiomas are rare benign proliferations of the lymphatic system and may be primary, occurring *de novo* in early childhood or sometimes later in life or secondary due to impaired lymphatic flow following destruction of the lymphatic channels due to disease, surgery or radiation. Secondary lymphangioma also called acquired lymphangioma or lymphangiectasis (AL) is a condition in which dilated superficial lymphatics develop after damage to previously normal lymphatics. AL is clinically and histologically indistinguishable from congenital lymphangioma circumscriptum. It is a difficult condition to treat as it tends to be chronic and recurrent, involving a large surface area, hence making surgical excision difficult. Superficial ablative techniques like CO_2_ lasers, electrotherapy and radiosurgery are palliative and usually followed by recurrences. A case of AL of the vulva is presented here treated by a novel combination technique of radiofrequency ablation with sclerotherapy with a successful outcome. Long-term follow-up was satisfactory.

## CASE REPORT

An 18-year-old female, from a rural background, presented with chronic, oozing, raised skin lesions on the vulva since the last five years. Discharge from the lesions was foul-smelling and associated with itching and burning. She had undergone treatment with electrocautery twice in the past, followed by recurrences each time. The last treatment was six months back. There was a history of bilateral swellings in the inguinal area at the age of nine years, which spontaneously ruptured and healed with scarring and was treated as tuberculous scrofuloderma.

**Figure 1:**
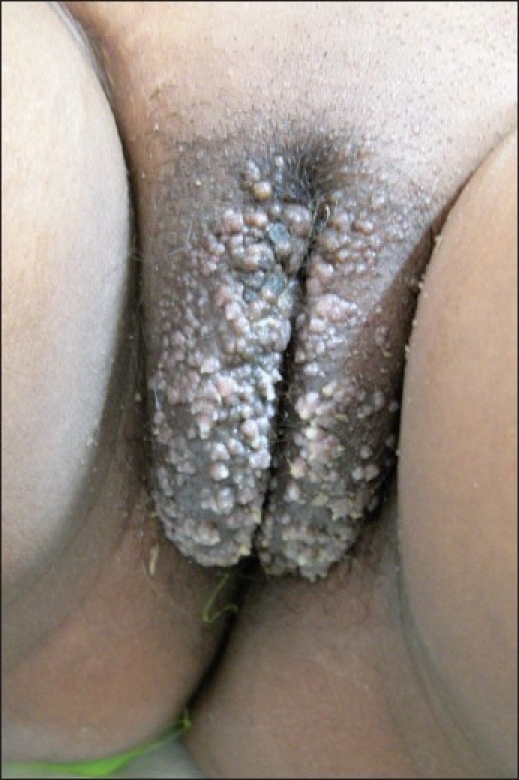
Lymphangiectasis of the vulva with deep-seated vesicles

**Figure 2:**
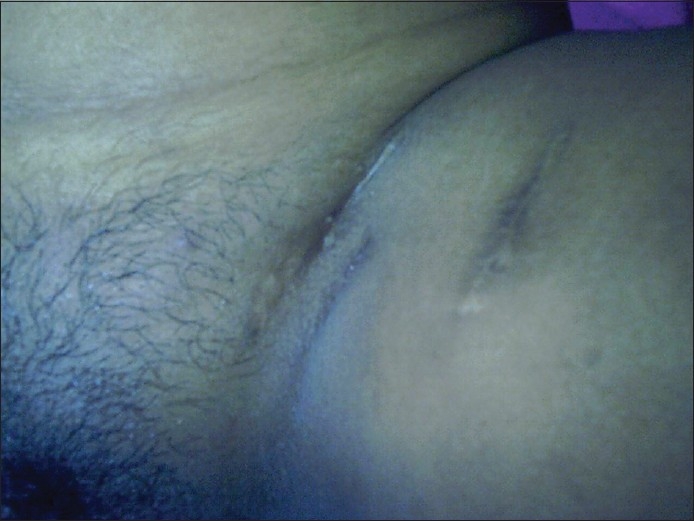
Inguinal scars in the left groin

On examination, multiple deep-seated vesicles containing clear, yellowish fluid were observed over both labia majora and labia minora, a few extending to the pubic area [Figure 1]. In addition, multiple scars were seen in the inguinal area [Figure 2].

Histopathological examination confirmed the diagnosis of lymphangiectasis. An abdominal ultrasound examination and computed tomography (CT) scan did not reveal any abnormality.

**Figure 3:**
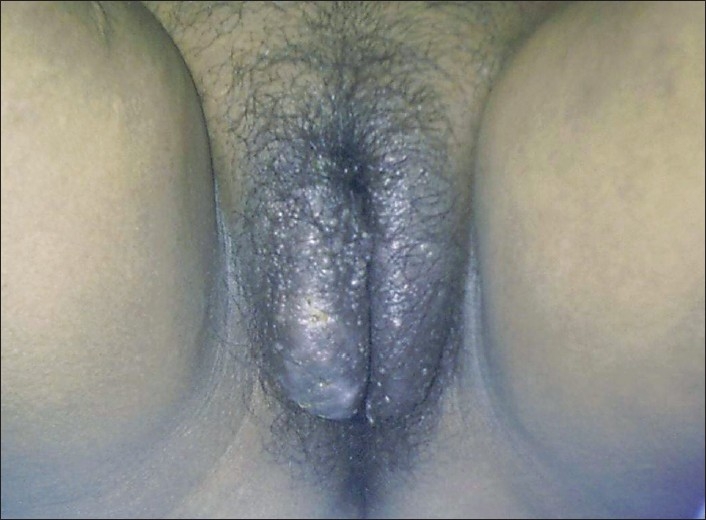
After two sessions of treatment

Radiofrequency ablation of the superficial vesicles was carried out under local anesthesia with 1% lignocaine using the Ellman-Surgitron® (EMC, USA) in the cut-coagulate mode at power 4 with a fine-needle electrode reaching up to the deep dermis. This was followed by sclerotherapy using 3% polidocanol intralesionally at the level of the subcutaneous tissue. Treatment was initiated on the left side and healing was complete in two weeks. It was then repeated on the right side. Sclerotherapy with 3% polidocanol solution was repeated monthly for three more sessions to prevent recurrence [Figure 3]. The patient was closely followed for recurrences and an additional treatment with 3% polidocanol was administered at three months after the last sitting as maintenance. There was no recurrence and patient was symptom-free after two years of follow-up.

## DISCUSSION

AL is an uncommon disorder and can occur in conditions associated with destruction of lymph nodes such as infections like filariasis, tuberculosis, erysipelas, and lymphogranuloma venereum, Crohn's disease, surgical or radiotherapeutic procedures, trauma, female genital mutilation, keloids, scleroderma and lymphatic obstruction associated with neoplasia.[1–5] Vulval lymphangiectasis has been reported following surgery and radiotherapy for carcinoma of the cervix or vulva,[6] Crohn's disease of the vulva,[7] and tubercular inguinal lymphadenitis.[8–12] There have been only six previously reported cases of vulval lymphangiectasis following tuberculous lymphadenitis.[12] The most common presentation is the appearance of deep-seated vesicles filled with a clear fluid. Rarely, they may develop a hyperkeratotic appearance, often misdiagnosed and treated as viral warts. This variation in the morphology is due to a gradual tissue organization, probably enhanced by the presence of lymphoedema.[13] Vulval AL can be asymptomatic or associated with burning, pruritis or pain. It is unpleasant due to the chronic discharge and can be a cause of recurrent cellulitis of the vulva.

The traditional treatment has been surgical removal, which is frequently associated with relapses.[14,15] In addition, surgical excision may not always be possible when wide areas are involved that can lead to distortion of normal anatomy of the vulva. Other modalities of treatment include electrocautery, CO_2_ laser,[15,16] Er:YAG laser.[17] There has been a report of keloid formation following CO_2_ laser treatment.[18] However, these are palliative treatments as they treat only the superficial component but do not address the deeper component of the lesions. Sclerotherapy alone is useful as it causes fibrosis of the deeper lymphatic cisterns, but the superficial skin lesions take a long time to improve.[19] Sclerotherapy should be repeated as all the dilated lymphatics may not be treated in a single session. Complications that may be observed following sclerotherapy include immediate transient swelling of the treated area, pain and ulceration if the sclerosant is injected too superficially in the dermis. Scarring and induration of the treated area may occur. These complications were not observed in our patient.

Hence this unique combination technique of radiofrequency ablation of the superficial lesions with sclerotherapy of the deeper lesions is synergistic and gives quicker results with acceptable cosmetic outcome. It has not been previously reported.

## CONCLUSION

The combination technique of radiofrequency ablation with sclerotherapy is a synergistic technique that is simple, efficacious and cost-effective with long-term satisfactory outcome, particularly in extensive, difficult to treat lymphangiectasis.
